# Factors Influencing Individuals' Decision-Making Regarding Hematopoietic Stem Cell Donation: A Cross-Sectional Study in Saudi Arabia

**DOI:** 10.7759/cureus.46015

**Published:** 2023-09-26

**Authors:** Renad S AlSubaie, Yousef A Alhamaid, Rahaf S Alali, Musawi A Altaha, Ahad A Aldalbahi, Sayed Ibrahim Ali

**Affiliations:** 1 Family and Community Medicine, College of Medicine, King Faisal University, Al-Ahsa, SAU

**Keywords:** transplantation, saudi arabia, donation, stem cell, decision-making

## Abstract

Introduction: Hematopoietic stem cell transplantation (HSCT) is a vital medical intervention for treating various conditions. The preferred methods, i.e., bone marrow transplantation and peripheral blood stem cell transplantation, have saved lives and attracted attention. Saudi Arabia, with a high sickle cell disease and leukemia incidence, faces the challenge of matching donors for HSCT. Factors like knowledge, attitudes, cultural beliefs, and access to information impact donation decisions.

Methods: In May 2023, a cross-sectional online survey was conducted in Saudi Arabia, targeting the general population. Data were collected through an online questionnaire, analyzing demographics, knowledge, attitudes, and factors influencing donation intention.

Results: Demographic analysis showed that females, younger individuals (18-25 years), those with higher education, and healthcare workers had better knowledge. Attitudes toward donation varied: 42.4% were willing to donate, while 57.6% were not. Psychological barriers, health concerns, pain, and inadequate knowledge influenced donation reluctance. Of the participants, 3.5% were registered stem cell donors, with 58.8% expressing willingness but not registered. Donors' intent was influenced by family members' need for transplants and knowledge. A majority (56.6%) supported employer support for health programs, while 65.7% believed government funding should assist donors.

Conclusion: HSCT is vital in treating diseases like sickle cell and leukemia in Saudi Arabia. While many recognize its importance, knowledge gaps about its specifics and donation deter potential donors. Enhanced awareness campaigns and support from employers and the government could increase donor registrations.

## Introduction

Hematopoietic stem cell transplantation (HSCT) represents a crucial medical intervention that holds the potential to save lives while mitigating a range of medical conditions, including leukemia, myelodysplastic syndromes, sickle cell anemia, thalassemia, severe combined immunodeficiency, and specific malignancies [1,2]. The two primary methods employed for HSCT are bone marrow transplantation (BMT) and peripheral blood stem cell transplantation (PBSCT) [3]. In approximately 75% of cases, PBSCT has become the preferred source of stem cells, supplanting BMT in recent years [3]. Due to the favorable outcomes achieved in treating particular severe medical conditions, stem cell research, therapy, and donation have captured the attention of the scientific community, healthcare organizations, and patients [4]. Saudi Arabia has the highest incidence of sickle cell disease among all Middle Eastern countries, with the majority of cases being reported in the eastern part of the country, followed by the southwestern region [5]. Despite the availability of stem cell treatment in Saudi Arabia since the late 1980s, securing a suitable match for transplantation remains a significant obstacle in stem cell therapy. As a result, a considerable number of patients are still on the waiting list for HSCT [6-8]. It is estimated that 60% of children and 30% of adults in need of HSCT cannot find compatible donors, highlighting the pressing need for alternative solutions in this field [6-8]. Obtaining the right match for HSCT has been a major challenge that has caught attention worldwide [9]. On the national level, a network of more than 12 million potential donors was founded by the National Marrow Donor Program (NMDP) in 1986 through Be The Match Registry, which facilitates the connection of patients with a human leukocyte antigen-matched donor [9]. On the local level, Saudi Arabia established the Saudi Stem Cell Donor Registry (SSCDR) and National Centre for Stem Cell Technology (NCSCT) in 2011 and 2014, respectively, under King Abdullah International Medical Research Center (KAIMRC)-National Guard Health Affairs; their main role is creating a database of individuals to donate their hematopoietic stem cells (HSCs) [10]. According to the official website of SSCDR, a total of 79,742 individuals have donated their HSCs through the registry [10]. Numerous studies have identified various factors that are linked to individuals' willingness to donate HSCs. These factors include insufficient knowledge about HSCT, confidence in the healthcare system, concerns about premature mortality, religious beliefs, discriminatory attitudes, family attitudes, apprehension about pain, and complications, among others [11-15]. On the ethical side, medical professionals oppose embryonic stem-cell research involving fetal destruction, understand the requirement of consent and ethical approval for research in this area, and support the need to regularize stem-cell-based therapy in Saudi Arabia [16]. As HSC donor registration is entirely optional in most nations, it is crucial to explore the elements that influence the intention to donate HSCs. Our study aims to investigate the factors that influence individuals' decision-making regarding HSC donation, including knowledge, attitudes, perceptions, and access to information and health care.

## Materials and methods

Methods

A cross-sectional online questionnaire-based study was conducted in Saudi Arabia from May 1 to June 20, 2023, using an online questionnaire to collect data from the Saudi population across different regions (central, southern, eastern, western, and northern). The study targeted the general population of Saudi Arabia, which harbors almost 34 million inhabitants [17], and a self-administered questionnaire was used to collect the data. Participants were provided with informed consent before filling out the questionnaire, which was distributed electronically via Google Forms (Google, Mountain View, CA). The data collected were entered into Microsoft Excel (Microsoft Corporation, Redmond, WA) and analyzed using the Statistical Package for the Social Sciences (SPSS) software (IBM Corp., Armonk, NY). Age was reported as mean ± SD for continuous variables, while gender was described using frequencies and percentages for categorical variables.

Inclusion criteria

The general population of Saudi Arabian citizens from both genders who agreed to participate in the study were included in this study.

Exclusion criteria

Participants who did not fill out the whole questionnaire, and those aged less than 18 years were excluded from the study.

Sampling technique and sample size

OpenEpi® version 3.0 software was used to estimate our sample size, which is representative of the Saudi Arabian population of 34 million. The representative sample size required was determined to be 385, with a margin error of 5% and a confidence level of 95%. However, we aimed to obtain a larger sample size than calculated to overcome potential exclusions. To achieve this, we used non-probability convenience sampling techniques. Descriptive statistics were performed for the collected data. Both the chi-square and t-test were performed using SPSS v.23 with the alpha criterion for the p-value set at 0.05.

Data collection instruments and procedures

The study questionnaire was developed by reviewing recent literature and questionnaires used in previous studies [13,18]. The survey was distributed based on data collectors and investigators’ networks. The questionnaire has seven sections. The first section includes demographic information such as age, gender, marital status, occupation, highest education level, having children, regular blood donor, a relative or a friend who is a registered HSC donor, a relative or a friend who needs bone marrow transplantation, and discussions with family about tissues and organs donation. The second section covered the preferred way of delivering information about stem cell donation. The third section assesses the knowledge of HSCT. The fourth section deals with factors influencing HSC donation intention. The fifth section was meant to find out the concerns of those unwilling to donate. The sixth section covered the behavior in the form of the willingness to be a stem cell donor for unrelated patients. The seventh section assesses the public support toward HSCT. The research excluded any answers that did not include informed consent and any incomplete responses from participants. The data were mainly gathered within the first two weeks of May 2023.

The first page of the questionnaire was designated for informed consent. An electronic survey using Google Forms was utilized and shared on various social media platforms like WhatsApp, Twitter, and Telegram. To ensure that the study criteria were met, the Google Forms feature "required to proceed" was used. Responses that did not include informed consent or were incomplete were excluded from the study.

Ethical consideration

All information was confidential, was only used for scientific research, and adhered to ethical guidelines for research involving human subjects. Ethical approval for conducting this study was obtained from the ethical approval committee at King Faisal University (reference number: KFU-REC-2023-MAY-ETHICS822). Participation in this research was voluntary and optional with informed consent on the first page. The data analysis and publication process did not require any identifiable personal data. The ethical approval of the study was obtained before data collection began.

## Results

Table 1 reveals that the majority of participants are female, constituting 66.4% of the group. The largest age group falls within the 18-25 years old category, comprising 49.9% of the sample. Additionally, individuals between 26 and 40 years old account for 24.0%, demonstrating a substantial presence of young and middle-aged adults. However, the representation of individuals above 60 years old is relatively low at 1.6%. The regional distribution highlights that the western region has the highest representation at 31.5%, closely followed by the eastern region at 29.2%. In contrast, the northern region is the least represented with only 2.0% of the participants belonging to this area.

**Table 1:** Sociodemographic variables

		n	%
Gender	Male	522	33.6%
	Female	1031	66.4%
Age	Less than 18 years old	86	5.5%
	18-25 years old	775	49.9%
	26-40 years old	373	24.0%
	41-60 years old	294	18.9%
	More than 60 years old	25	1.6%
Region	Eastern	454	29.2%
	Western	489	31.5%
	Southern	441	28.4%
	Northern	31	2.0%
	Central	138	8.9%
Marital status	Single	890	57.3%
	Married	607	39.1%
	Divorced	43	2.8%
	Widow	13	0.8%
Highest educational level	Uneducated	57	3.7%
	Primary school	6	0.4%
	High school	551	35.5%
	University level	839	54.0%
	Postgraduate level	100	6.4%
Occupation	Healthcare worker	95	6.1%
	Non-healthcare worker	440	28.3%
	Student	706	45.5%
	Freelancer	72	4.6%
	Housewife	152	9.8%
	Looking for a job	88	5.7%
Having children	Yes	556	35.8%
	No	997	64.2%
Regular blood donor	Yes	376	24.2%
	No	1177	75.8%
Do you have a relative in need of a hematopoietic stem cell transplant?	Yes	151	9.7%
	No	1402	90.3%
Do you know anyone in need of a hematopoietic stem cell transplant?	Yes	196	12.6%
	No	1357	87.4%

Regarding marital status, the majority of participants are single, accounting for 57.3% of the group. Married individuals make up a significant portion at 39.1%, while divorced individuals and widows have comparatively lower percentages at 2.8% and 0.8%, respectively. Educational attainment among participants reveals that the majority, comprising 54.0%, have reached the university level. High school graduates account for 35.5%, while those with a postgraduate level of education represent 6.4% of the sample. The table also sheds light on the occupations of the participants. Notably, students form the largest occupational group at 45.5%, indicating a significant presence of students within the surveyed population. Healthcare workers constitute a smaller proportion at 6.1%, while non-healthcare workers have a higher representation of 28.3%. Examining the aspect of having children, the data reveal that 35.8% of participants have children, while the majority, comprising 64.2%, do not. The table further highlights the prevalence of regular blood donors, with 24.2% of participants reporting as such, while the majority, at 75.8%, do not regularly donate blood. Finally, the data touch upon the need for HSCT. Only 9.7% of participants report having a relative in need of a transplant, while a slightly higher percentage (12.6%) knows someone who requires this medical procedure.

Table 2 provides valuable insights into the respondents' knowledge and awareness of HSCT and related topics. The findings indicate that while a significant majority of respondents (74.0%) have heard about HSCT before, there is still a considerable portion (19.7%) who are unfamiliar with the procedure. This highlights the need for further education and awareness campaigns to ensure broader knowledge dissemination about HSCT.

**Table 2:** Knowledge items frequencies and percentages

Knowledge items		n	%
Have you heard about hematopoietic stem cell transplants before?	Yes	1149	74.0%
	No	306	19.7%
	I don’t know	98	6.3%
Can hematopoietic stem cells used as a treatment?	Yes	1102	71.0%
	No	82	5.3%
	I don’t know	369	23.8%
What do you know about hematopoietic stem cell transplants?	A blood transfusion process	94	6.1%
	A surgery where bones are cut off and given to another person	108	7.0%
	Cells collected from suitable donors and injected into the recipient’s body	974	62.7%
	Nothing	377	24.3%
Can a hematopoietic stem cell transplant result in severe complications?	Yes	263	16.9%
	No	203	13.1%
	I don’t know	1087	70.0%
Will there be any visible scars after stem cell donation?	Yes	169	10.9%
	No	362	23.3%
	I don’t know	1022	65.8%
Are you aware of local or international stem cell donor registry?	Yes	126	8.1%
	No	1224	78.8%
	I don’t know	203	13.1%
Can registered stem cell donors withdraw their registration?	Yes	367	23.6%
	No	163	10.5%
	I don’t know	1023	65.9%

Moreover, the perception of HSCT as a treatment option is somewhat positive, with 71.0% of respondents acknowledging its potential. However, it is noteworthy that a notable percentage (23.8%) expressed uncertainty, indicating the need for more information to foster a better understanding of HSCT's efficacy and limitations as a treatment modality. When examining the respondents' knowledge about the HSCT process, it is encouraging that the majority (62.7%) correctly identified that it involves collecting cells from suitable donors and injecting them into the recipient's body. However, the fact that 24.3% of respondents knew nothing about the process underscores the importance of public education initiatives to ensure a better understanding of the treatment procedure. The survey reveals a significant knowledge gap concerning the potential complications associated with HSCT. The fact that 70.0% of respondents were unsure about the existence of severe complications suggests a lack of awareness in this area. Enhancing public knowledge about the possible risks and side effects is crucial to support informed decision-making and better patient outcomes.

Furthermore, the table highlights a lack of understanding regarding the physical consequences of stem cell donation. The majority of respondents (65.8%) expressed uncertainty about the presence of visible scarring, suggesting the need for improved communication and education about the potential outcomes and recovery processes associated with stem cell donation. The low awareness of local or international stem cell donor registries is another significant finding from the table. With 78.8% of respondents indicating their lack of awareness, it emphasizes the importance of raising public knowledge about these registries. Encouraging individuals to join and support such initiatives can significantly impact the availability of suitable donors for HSCT and other stem cell therapies.

Lastly, the respondents' uncertainty regarding the ability of registered stem cell donors to withdraw their registration reflects the need for clearer guidelines and information regarding the withdrawal process. Providing transparent and accessible information about registration procedures and the freedom to withdraw will help individuals make informed decisions and alleviate any concerns about commitment.

Table 3 presents a range of attitudes and opinions regarding bone marrow donation, stem cell donation, and related topics. On one hand, a majority of respondents (57.6%) expressed a reluctance to donate bone marrow or undergo a transplant, indicating a hesitancy toward such procedures. However, it is worth noting that a significant proportion (42.4%) displayed a willingness to donate or undergo a transplant, suggesting a level of openness and altruism within the surveyed group. In terms of stem cell donor registration, the table reveals that a small percentage (3.5%) had already registered as donors, demonstrating proactive engagement with the cause. Furthermore, a substantial portion (58.8%) expressed a willingness to register in the future, indicating a potential for an increased stem cell donor pool. However, it is important to acknowledge that a significant number of respondents (37.7%) stated that they were not willing to be registered as stem cell donors, suggesting various personal reasons or concerns surrounding the donation process. Another aspect highlighted in the table is the presence of discussions surrounding tissue and organ donation within families. Although a minority (22.0%) reported having engaged in such conversations, the majority (78.0%) indicated that no such discussions had taken place. This observation suggests a need for increased awareness and dialogue within families about the importance of tissue and organ donation, potentially fostering a more supportive environment for future donations.

**Table 3:** Attitude items distribution

Attitude items		n	%
Would you like to donate bone marrow, undergo a bone marrow transplant, or allow your child to receive or donate?	Yes	658	42.4%
	No	895	57.6%
Are you willing to be a stem cell donor for unrelated patients?	Yes, I have already registered	54	3.5%
	Yes, but I have not yet registered	913	58.8%
	No	586	37.7%
Discussions with family about tissue and organ donation	Yes	341	22.0%
	No	1212	78.0%
Attitudes toward hematopoietic stem cell donation	Willing to donate to family members only	230	14.8%
	Willing to donate to unrelated individuals	522	33.6%
	Willing to donate to family members and unrelated individuals	212	13.7%
	Unwilling to donate	589	37.9%
If you are the employer, would you agree to grant a special release for endorsed health programs or equivalent (e.g., stem cell donation) to your staff?	Strongly agree	390	25.1%
	Agree	489	31.5%
	No comment	583	37.5%
	Disagree	67	4.3%
	Strongly disagree	24	1.5%
Do you agree that the stem cell donors or their employers should be funded by the government?	Strongly agree	488	31.4%
	Agree	532	34.3%
	No comment	481	31.0%
	Disagree	34	2.2%
	Strongly disagree	18	1.2%

When examining attitudes toward HSC donation, the responses indicate a range of perspectives. A portion of participants (14.8%) expressed a willingness to donate HSCs exclusively to family members, while a larger percentage (33.6%) showed a willingness to donate to unrelated individuals. Additionally, a notable subset (13.7%) expressed a willingness to donate to both family members and unrelated individuals, showcasing a broader sense of altruism and inclusivity. However, it is important to acknowledge that a significant proportion (37.9%) indicated an unwillingness to donate, suggesting a need for further exploration of the underlying reasons and potential barriers to donation. In terms of employer support for health programs, a combined 56.6% of respondents either strongly agreed or agreed to grant special release for endorsed health programs or equivalent initiatives to their staff. This finding highlights a level of employer recognition and support for the importance of health programs, potentially fostering a more positive work environment and facilitating greater participation in such programs. Nevertheless, it is worth noting that a considerable proportion (37.5%) had no comment, which may suggest a lack of awareness or indecision regarding this matter.

Finally, the table presents opinions on government funding for stem cell donors and their employers. A significant portion (65.7%) either strongly agreed or agreed that the government should provide funding for stem cell donors, indicating a recognition of the financial implications and potential burden associated with donation. Conversely, a minority expressed disagreement (2.2%) or strong disagreement (1.2%) with this proposition, potentially suggesting differing perspectives on the role of government support in facilitating and incentivizing stem cell donation.

**Figure 1:**
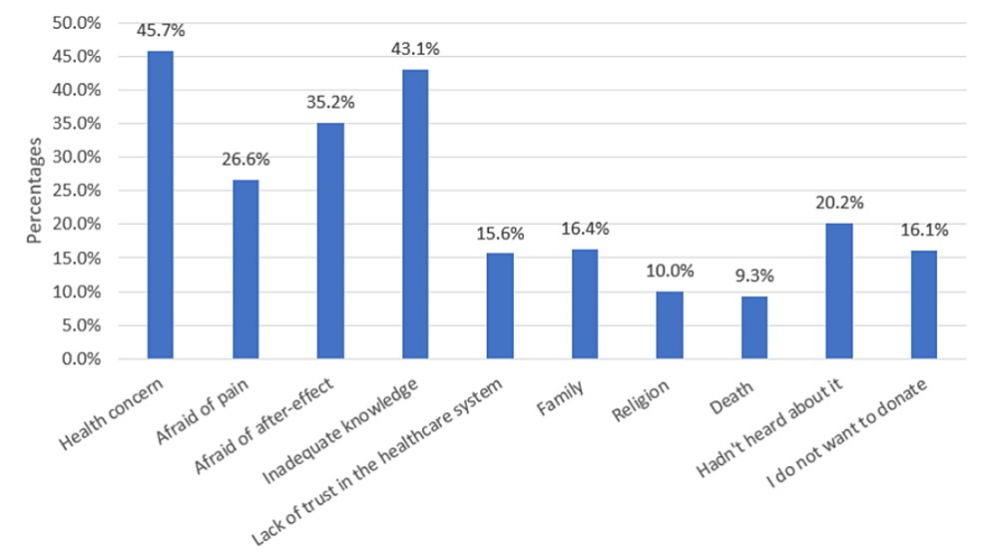
Factors contributing to resistance to donating hematopoietic stem cell

Figure 1 presents a visual representation of the primary factors influencing low donation rates among the surveyed population. The data clearly indicate that health concerns emerge as the most significant reason preventing individuals from donating, while fear of death appears to be the least inhibiting factor. The dominance of health concerns as the primary barrier to donation aligns with previous research highlighting the importance of personal health and well-being in decision-making processes. It underscores the need for health campaigns and donor education programs to address these concerns proactively. Such initiatives can focus on dispelling myths, providing accurate information on the safety of donation procedures, and highlighting the potential health benefits associated with altruistic acts of donation.

Conversely, the relatively lower impact of the fear of death as a deterrent to donation is an interesting finding. This suggests that individuals may be more open to considering donation, even when confronted with existential fears. It is worth noting that while health concerns and fear of death emerge as the most and least significant factors, respectively, other factors also play crucial roles in influencing donation behavior.

Finally, the figure underscores the importance of addressing health concerns as a central focus in campaigns aimed at improving donation rates.

**Figure 2:**
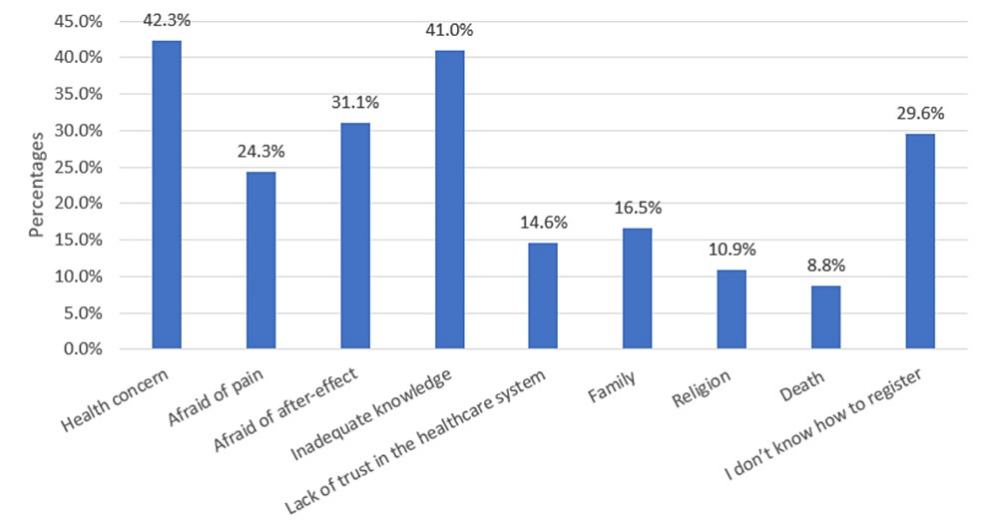
Barriers to hematopoietic stem cell donation registration

Figure 2 reveals a spectrum of barriers to HSC donation registration. The most significant hurdles are inadequate knowledge and health concerns, with a substantial portion of potential donors expressing reservations due to a lack of information and fears related to the donation process. On the other hand, the comparatively lower barriers include religious beliefs and concerns related to death. While these barriers affect a smaller percentage of potential donors, they are nonetheless important to address, necessitating a respectful approach that aligns donation efforts with diverse cultural and personal beliefs, as well as providing emotional support for those concerned about mortality. Finally, addressing these varied barriers comprehensively, with a focus on knowledge dissemination and alleviating health-related fears, while respecting individual beliefs and offering support, can encourage more individuals to consider HSC donation registration and potentially save lives.

Table 4 shows that a higher percentage of females possess good knowledge compared to males (p = 0.005). Additionally, the data indicate that knowledge tends to increase with age, with the highest percentage of good knowledge observed among individuals aged 18-25 years (p = 0.0001). Moreover, the table reveals that higher educational attainment is associated with a greater percentage of good knowledge. The statistically significant p-value of 0.000 underscores the importance of education in enhancing awareness and understanding of HSCT. Occupation also appears to influence knowledge levels, with healthcare workers demonstrating a higher percentage of good knowledge compared to non-healthcare workers (p = 0.013). Furthermore, individuals who have a relative or acquaintance in need of a transplant tend to possess a higher percentage of good knowledge. These findings, indicated by p-values of 0.002 and 0.000, respectively, highlight the potential impact of personal experiences on awareness and understanding of the topic.

**Table 4:** Association between sociodemographic data and knowledge

		Knowledge				p-value
		Poor		Good		
		n	%	n	%	
Gender	Male	227	37.9%	295	30.9%	0.005
	Female	372	62.1%	659	69.1%	
Age	Less than 18 years old	54	9.0%	32	3.4%	0.000
	18-25 years old	278	46.4%	497	52.1%	
	26-40 years old	144	24.0%	229	24.0%	
	41-60 years old	111	18.5%	183	19.2%	
	More than 60 years old	12	2.0%	13	1.4%	
Region	Eastern	170	28.4%	284	29.8%	0.246
	Western	178	29.7%	311	32.6%	
	Southern	174	29.0%	267	28.0%	
	Northern	16	2.7%	15	1.6%	
	Central	61	10.2%	77	8.1%	
Marital status	Single	337	56.3%	553	58.0%	0.065
	Married	247	41.2%	360	37.7%	
	Divorced	14	2.3%	29	3.0%	
	Widow	1	0.2%	12	1.3%	
Highest educational level	Uneducated	40	6.7%	17	1.8%	0.000
	Primary school	4	0.7%	2	0.2%	
	High school	234	39.1%	317	33.2%	
	University level	295	49.2%	544	57.0%	
	Postgraduate level	26	4.3%	74	7.8%	
Occupation	Healthcare worker	23	3.8%	72	7.5%	0.013
	Non-healthcare worker	171	28.5%	269	28.2%	
	Student	266	44.4%	440	46.1%	
	Freelancer	33	5.5%	39	4.1%	
	Housewife	71	11.9%	81	8.5%	
	Looking for a job	35	5.8%	53	5.6%	
Having children	Yes	214	35.7%	342	35.8%	0.961
	No	385	64.3%	612	64.2%	
Regular blood donor	Yes	133	22.2%	243	25.5%	0.143
	No	466	77.8%	711	74.5%	
Do you have a relative in need of a hematopoietic stem cell transplant?	Yes	41	6.8%	110	11.5%	0.002
	No	558	93.2%	844	88.5%	
Do you know anyone in need of a hematopoietic stem cell transplant?	Yes	44	7.3%	152	15.9%	0.000
	No	555	92.7%	802	84.1%	

On the other hand, factors such as region, marital status, having children, being a regular blood donor, and looking for a job do not demonstrate statistically significant associations with knowledge levels, as reflected by the respective p-values.

Table 5 shows that a significantly higher percentage of individuals with good knowledge are willing to donate compared to those with poor knowledge (p = 0.0001). Interestingly, a higher percentage of individuals who have already registered as donors or plan to register have good knowledge. Again, the p-value of 0.0001 indicates a significant association between knowledge and the willingness to be a donor. Results also reveal that individuals with good knowledge are more likely to have engaged in discussions with their family about donation compared to those with poor knowledge (p = 0.0001).

**Table 5:** Association between attitude items and knowledge

		Knowledge					p-value
		Poor		Good			
		n	%		n	%	
Would you like to donate bone marrow, undergo a bone marrow transplant, or allow your child to receive or donate?	Yes	151	25.2%		507	53.1%	0.000
	No	448	74.8%		447	46.9%	
Are you willing to be a stem cell donor for unrelated patients?	Yes, I have already registered	13	2.2%		41	4.3%	0.000
	Yes, but I have not yet registered	316	52.8%		597	62.6%	
	No	270	45.1%		316	33.1%	
Discussions with family about tissue and organ donation	Yes	84	14.0%		257	26.9%	0.000
	No	515	86.0%		697	73.1%	
Attitudes toward hematopoietic stem cell donation	Willing to donate to family members only	64	10.7%		166	17.4%	0.000
	Willing to donate to unrelated individuals	139	23.2%		383	40.1%	
	Willing to donate to family members and unrelated individuals	80	13.4%		132	13.8%	
	Unwilling to donate	316	52.8%		273	28.6%	
If you are the employer, would you agree to grant a special release for endorsed health programs or equivalent (e.g., stem cell donation) to your staff?	Strongly agree	97	16.2%		293	30.7%	0.000
	Agree	173	28.9%		316	33.1%	
	No comment	275	45.9%		308	32.3%	
	Disagree	41	6.8%		26	2.7%	
	Strongly disagree	13	2.2%		11	1.2%	
Do you agree that the stem cell donors or their employers should be funded by the government?	Strongly agree	122	20.4%		366	38.4%	0.000
	Agree	190	31.7%		342	35.8%	
	No comment	254	42.4%		227	23.8%	
	Disagree	22	3.7%		12	1.3%	
	Strongly disagree	11	1.8%		7	0.7%	

The next section focuses on attitudes toward HSC donation. The data suggest that individuals with good knowledge are more willing to donate to unrelated individuals compared to those with poor knowledge (p = 0.0001). The following section examines the agreement of employers to grant special releases for endorsed health programs, including stem cell donation, to their staff. The table shows that a higher percentage of employers who strongly agree or agree with this idea have good knowledge (p = 0.0001).

Lastly, the table presents data on the agreement regarding government funding for stem cell donors or their employers. Individuals with good knowledge are more likely to strongly agree or agree with government funding (p = 0.0001).

## Discussion

In Saudi Arabia, although the SSCDR and cord blood banking units have seen an increase in the number of registered volunteer donors, there is still a need for more potential donors to meet the growing demand for HSCT among patients [19]. Notably, between 1984 and 2016, a total of 6184 HSCTs were performed in Saudi Arabia, primarily for the treatment of malignancies like acute lymphoblastic leukemia, acute myelogenous leukemia, and chronic myelocytic leukemia. HSCT is considered the most effective therapy for life-threatening blood disorders [20]. The current study has successfully identified crucial factors influencing the willingness of individuals to donate HSCs, as well as the reasons that hinder citizens from participating.

Regarding HSCT knowledge, our results demonstrated that 74.0% of participants were familiar with and had heard about HSCT before, and 62.7% accurately defined it. However, nearly 70.0% of individuals were uncertain about potential severe complications or visible scars resulting from stem cell donation. Similarly, a study by Adediran et al. [20] found that almost 65% of participants had awareness of HSCT, and their level of knowledge was significantly linked to a more positive attitude. In our current study, we also observed a significant association between gender and knowledge, with females demonstrating a higher level of understanding (69.1% having good knowledge) compared to males (30.9%) (Table 4). This finding aligns with another study conducted in Riyadh [21], which reported that being female was significantly linked to a better understanding of HSCT.

Our study demonstrated that the majority of the participants were hesitant to undergo transplant and donate bone marrow (57.6%). However, the rest of the participants (42.4%) were interested in donating their bone marrow. Donation barriers were examined to gain a deeper understanding of the reasons behind individuals' reluctance to donate HSCs, including to their own family members. The findings revealed that psychological barriers such as "health concerns," "fear of pain," "worry about after-effects," and "inadequate knowledge" were significant factors contributing to resistance to donating HSCs (Figure 1), whether to family or unrelated individuals. These concerns of pain, after-effects, and lack of knowledge were also identified in previous studies [22-25]. Our study suggests that providing better education on HSCT may assist hesitant individuals in overcoming these fears. Interestingly, religion consistently emerged as the least important factor influencing the unwillingness to donate to family members or unrelated individuals alongside the fear of death, which aligns with the findings of a previous study [24].

The attitude toward HSC donation is not consistent and is influenced by the specific recipient of the donation. Our research has uncovered significant differences in individuals' willingness to donate based on whether the intended recipient is a family member or an unrelated individual. Surprisingly, a small percentage of participants (33.6%) expressed a willingness to donate to unrelated individuals. Additionally, only a few participants (13.7%) were willing to donate to family members and unrelated individuals. This contrasts with the results of a previous study conducted by Abdrbo et al. [26], which demonstrated a higher willingness (47.2%) to donate to both family members and unrelated individuals. Furthermore, according to Narayanan et al., 72% of participants reported being motivated to donate their stem cells to any patient [27]. This can be attributed to the widely accepted knowledge that individuals generally exhibit stronger empathetic responses and engage in more helpful behaviors toward their close relatives [28].

Our study revealed that a mere 3.5% of participants were registered as stem cell donors. Interestingly, a significant proportion (58.8%) expressed the desire to donate, but they were not enrolled in a stem cell donor registry. Similarly, Abdrbo et al. [26] showed how 67.7% of participants expressed enthusiasm for donation, yet they were not registered as donors. Surprisingly, only 5.2% of participants in their study were actually registered. This indicates that a large number of individuals have the intention to donate but have not taken action yet. We believe that this is due to their lack of familiarity with the registration process, as evidenced by nearly 500 participants who expressed uncertainty about how to donate (Figure 2).

According to a study conducted by Kita et al. [29], it was discovered that individuals who engage in conversations about organ and tissue donation with their family members are more likely to register for HSC donation. Regrettably, in our study, we observed that 78.0% of participants have not discussed HSC donation with their families. This could be ascribed to a lack of individuals' motivation toward engaging in the act of donation.

Following HSCT procedures, donors typically require a recovery period of approximately seven to 10 days, which can lead to financial loss for both the donors themselves and their employers [29]. This financial impact has the potential to discourage individuals from participating in HSC donations [29]. Consequently, our study focused on assessing the level of support of the population for initiatives such as the "time-off-work scheme" or similar government-funded programs that aim to provide financial compensation to donors and employers [29]. The findings demonstrated that over half of the participants expressed support for paid leaves for employees involved in HSC donations, while up to 60% agreed that some form of government funding should be accessible to employers.

Limitations

The study conducted via a cross-sectional online survey may exhibit selection bias as not all demographic groups have equal online access or proficiency, potentially leading to underrepresentation, especially among older populations. The findings, specific to Saudi Arabia, might not be generalizable to different cultural or economic contexts. Lastly, if cultural nuances were not meticulously factored into the survey design, some questions might be subject to misinterpretation. Future research might benefit from a mixed-methods approach and stratified sampling to ensure comprehensive and nuanced findings.

## Conclusions

HSCT serves as a crucial remedy for severe medical conditions. In Saudi Arabia, there is a pressing demand for donors due to prevalent diseases such as sickle cell and leukemia. Our research indicates that while there is general awareness about HSCT, many lack in-depth knowledge, including potential side effects and the registration process. Despite the willingness of many to donate, barriers such as anxiety, health apprehensions, and knowledge gaps deter them. Enhancing public understanding through awareness campaigns, fostering family discussions on donation, seeking employer backing, and obtaining government assistance can pave the way to boosting donor numbers in Saudi Arabia.
